# Effects of data preprocessing on results of the epidemiological analysis of coronary heart disease and behaviour-related risk factors

**DOI:** 10.1080/07853890.2021.1921838

**Published:** 2021-06-23

**Authors:** Ari Voutilainen, Christina Brester, Mikko Kolehmainen, Tomi-Pekka Tuomainen

**Affiliations:** a Institute of Public Health and Clinical Nutrition, University of Eastern Finland, Kuopio, Finland; b Department of Environmental and Biological Sciences, University of Eastern Finland, Kuopio, Finland

**Keywords:** Categorical covariate, continuous covariate, coronary heart disease, exclusion criterion, outcome sensitivity

## Abstract

**Background:**

We carried out this study to demonstrate the effects of outcome sensitivity, participant exclusions, and covariate manipulations on results of the epidemiological analysis of coronary heart disease (CHD) and its behaviour-related risk factors.

**Material and methods:**

Our study population consisted of 1592 54-year-old men, who participated in the Kuopio Ischaemic Heart Disease Risk Factor (KIHD) Study. We used the Cox proportional-hazards model to predict the hazard of CHD and applied different sets of outcomes concerning outcome sensitivity and data preprocessing procedures regarding participant exclusions and covariate manipulations.

**Results:**

The mean follow-up time was 23 years, and 730 men received the CHD diagnosis. Cox regressions based on data with no participant exclusions most often discovered statistically significant associations. Loose inclusion criteria for study participants with any CVD during the follow-up and strict exclusion criteria for participants with no CVD were best in discovering the associations between risk factors and CHD. Outcome sensitivity affected the associations, whereas the covariate type, continuous or categorical, did not.

**Conclusions:**

This study suggests that excluding study participants who are not disease-free at baseline is probably unnecessary for epidemiological analyses. Epidemiological research reports should present results based on no data exclusions together with results based on reasoned exclusions.

## Introduction

Typically, epidemiologic research produces at least partly contradictory results. Some reasons explaining this incoherence i.e. unexpectedly large variations in results across closely related studies, are only indirectly related to research, such as clinical factors and healthcare systems. Many reasons, however, originate from study designs, methodological choices, concept definitions, and observed data [1,2]. Reasons related to datasets include at least differences in sample size and representativeness of covariates. In prospective cohort studies, also the length of follow-up with respect to age at baseline amongst study participants, as in the risk of coronary heart disease (CHD) associated with high levels of C-reactive protein [3], and possible competing events affect the interpretation of study results [4].

Research regarding the epidemiologic relationship between CHD and risk factors has received a consensus during the past decades. There are six undisputable behaviour-related risk factors for CHD: tobacco smoke [5], overweight [6], physical inactivity [7], hypertension [8], diabetes [9], and hypercholesterolaemia [10].

Other behaviour-related factors, such as alcohol consumption and stress also may increase the risk for CHD, but their associations with CHD vary across studies. The association between alcohol and CHD is nonlinear [11], and stress is a symptom of different conditions, such as psychosocial aspects of work [12], which may or may not be associated with the risk of CHD. Yet other risk factors of CHD that at least indirectly relate to behaviour through diet are homocysteine, fibrinogen, and inflammation [13]. Moreover, there may be a weak association between iron status and CHD [14].

In addition to the behaviour-related factors, non-modifiable factors including age, male gender, genetics, and a family history of CHD increase the risk for CHD [13,15,16]. Differences between men and women regarding the risk of CHD relate mainly to oestrogens and, thus, premenopausal women [13]. The role played by personality in the development of CHD is controversial [17].

The purpose of this study was to demonstrate the effects of data exclusions, outcome variable selection, and covariate manipulations on the interpretation of the epidemiologic relationship between CHD and its traditional risk factors. These are predominantly subjective researcher-related actions unlike more technical questions, such as whether to consider competing events in statistical analyses or whether to use non-conventional statistical methods, such as neural networks [18], to deal with data-related matters. As a result of this study, we expected a combination of outcome variable selection, participant exclusion, and covariate manipulation procedures that best discovers presumable associations between CHD and risk factors.

## Material and methods

### Material

Men, *n* = 1592, from the Kuopio Ischaemic Heart Disease Risk Factor (KIHD) Study served as a study material. The KIHD Study is an ongoing prospective cohort study originally established to discover previously unestablished reasons for the extremely high AMI prevalence among eastern Finnish men [19]. To control the effect of age on CHD we selected men representing the same age cohort, 54-year-old at baseline between March 1984 and December 1989. Briefly, 778 of them had one or more CVDs at baseline based on self-reports to the question: Has your doctor told you that you have ‘the name of CVD’, and 1181 of them were diagnosed, during an inpatient special health care admission, as having CVDs, ICD-10 codes I00-I99 [20], by the end of 2017. Moreover, 381 men used medication for hypertension, 77 had insulin or non-insulin treated diabetes, and nine used medication for hypercholesterolaemia at baseline. The mean (SD) follow-up time was 23.4 (9.3) years. Table 1 presents study participants’ baseline characteristics with respect to variables used as exclusion criteria, covariates, and conditions and events diagnosed during the follow-up. All KIHD participants had given written informed consent, and the ethical committee of the Kuopio University had approved the KIHD Study (December 1, 1983). In 1980s, the committee did not necessarily provide study numbers but identified studies by date.

**Table 1.** Baseline characteristics (the total column) and numbers of study participants with the following conditions diagnosed during the follow-up: any cardiovascular disease (CVD), coronary heart disease (CHD), a myocardial infarction (MI) or unstable angina (UA), and a fatal acute myocardial infarction (AMI).

		Conditions and events diagnosed during the follow-up			
Characteristic	Total	CVD	CHD	MI or UA	AMI
*n*	1592	1181	730	502	136
CVD, excluding hypertension, *n* (%)	672 (42)	542 (46)	375 (51)	260 (52)	83 (61)
Use of hypertension medication, *n* (%)	381 (24)	318 (27)	240 (33)	168 (34)	58 (43)
Diabetes, *n* (%)	77 (4.8)	61 (5.2)	47 (6.4)	41 (8.2)	10 (7.4)
Use of cholesterol medication, *n* (%)	9 (0.6)	9 (0.8)	8 (1.1)	5 (1.0)	2 (1.5)
Cigarette-year^a^, mean (SD)	336 (392)	339 (402)	356 (403)	387 (428)	431 (478)
Never-smokers, *n* (%)	517 (33)	377 (32)	217 (30)	133 (27)	34 (25)
Former smokers, *n* (%)	572 (36)	443 (38)	274 (38)	196 (39)	42 (31)
Current smokers, *n* (%)	503 (32)	361 (31)	239 (33)	173 (35)	60 (44)
Alcohol, grams/week, mean (SD)	71 (141)	66 (105)	62 (92)	64 (96)	75 (100)
No risk, ≤ 1 portion/week, *n* (%)	604 (38)	449 (38)	285 (39)	186 (37)	53 (39)
Moderate risk, ≤ 3 portions/day, *n* (%)	906 (57)	676 (57)	415 (57)	295 (59)	75 (55)
High risk, > 3 portions/day, *n* (%)	82 (5.2)	56 (4.7)	30 (4.1)	21 (4.2)	8 (5.9)
Body Mass Index (BMI), mean (SD)	27 (3.7)	27 (3.7)	27 (3.7)	27 (3.7)	28 (4.5)
Normal weight, BM*I* < 25.0 kg/m^2^, *n* (%)	480 (30)	324 (27)	183 (25)	118 (24)	35 (26)
Overweight, BMI 25.0 − 29.9 kg/m^2^, *n* (%)	830 (52)	622 (53)	400 (55)	278 (55)	60 (44)
Obese, BM*I* ≥ 30.0, kg/m^2^, *n* (%)	282 (18)	235 (20)	147 (20)	106 (21)	41 (30)
Physical activity^b^, kcal/day, mean (SD)	2380 (899)	2377 (886)	2349 (888)	2326 (877)	2381 (987)
Moderate, PAL^c^ < 2.00, *n* (%)	293 (18)	222 (19)	144 (20)	101 (20)	29 (21)
Vigorous, PAL 2.00 − 2.40, *n* (%)	507 (32)	377 (32)	236 (33)	165 (33)	41 (30)
Extreme, PA*L* > 2.40, *n* (%)	774 (49)	571 (49)	341 (47)	229 (46)	65 (48)
No data available, *n*	18	11	9	7	1
Systolic blood pressure, mean (SD)	136 (18)	137 (18)	136 (18)	137 (19)	139 (18)
Desirable, < 120 mmHg, *n* (%)	269 (17)	186 (16)	122 (17)	89 (18)	17 (13)
Borderline, 120 − 139 mmHg, *n* (%)	763 (48)	552 (47)	331 (45)	213 (42)	61 (45)
High, ≥ 140 mmHg, *n* (%)	560 (35)	443 (38)	277 (38)	200 (40)	58 (43)
Fasting blood glucose, mean (SD)	4.8 (1.2)	4.9 (1.3)	4.9 (1.4)	5.0 (1.5)	5.1 (1.6)
Desirable, < 5.6 mmol/L, *n* (%)	1435 (90)	1056 (89)	636 (87)	432 (86)	112 (82)
Borderline, 5.6 − 6.9 mmol/L, *n* (%)	96 (6.0)	71 (6.0)	53 (7.3)	40 (8.0)	12 (8.8)
High, > 6.9 mmol/L, *n* (%)	61 (3.8)	54 (4.6)	41 (5.6)	30 (6.0)	12 (8.8)
Serum total cholesterol, mean (SD)	6.0 (1.1)	6.0 (1.1)	6.1 (1.2)	6.2 (1.2)	6.3 (1.2)
Desirable, < 5.2 mmol/L, *n* (%)	383 (24)	270 (23)	151 (21)	93 (19)	25 (19)
Borderline, 5.2 − 6.2 mmol/L, *n* (%)	607 (38)	443 (38)	274 (38)	190 (38)	46 (34)
High, > 6.2 mmol/L, *n* (%)	602 (38)	468 (40)	305 (42)	219 (44)	65 (48)

^a^ Cigarettes per day times years of smoking.

^b^ Total energy expenditure (TEE) minus basal energy expenditure (BEE).

^c^ Physical activity level, TEE divided by BEE.

### Outcome variables

The KIHD Study includes annually updated data from the Care Register for Health Care of the Finnish Institute for Health and Welfare regarding diagnoses given during special health care admissions (License THL/93/5.05.00/2013) and from the Causes of Death Register of the Statistics Finland (License TK-53-1770-16). To study the effects of outcome sensitivity on model results we constructed four different outcome variables based on these register linkages. The first outcome was ‘CVD’ referring to ICD 10 codes I00 − I99. The second outcome was ‘CHD’ referring to codes I20 − I25. The third outcome was ‘MI or UA’ and it referred to codes I20.0 and I21 − I22. The fourth outcome ‘a fatal AMI’ referred to as I21.

### Covariates

First, we selected the most common risk factors of CHD based on literature and, second, we searched variables that represent these risk factors from the KIHD Study database. The chosen risk factors were smoking, obesity, physical inactivity, hypertension, diabetes, and hypercholesterolaemia. Hajar [21], for example, summaries the association between these six risk factors and CHD. In addition to the indisputable risk factors of CHD, we included alcohol consumption as a covariate in the analyses. Alcohol, in general, increases mortality and morbidity [22], but the association between alcohol consumption and CHD is visualized by a J-shaped curve; light-to-moderate drinking acts as a protective factor, whereas heavy drinking increases the risk of CHD [11]. We expected that our analyses at best would demonstrate this nonlinear relationship between alcohol consumption and CHD.

In the KIHD Study, participants self-reported their smoking behaviour, alcohol consumption, and physical activity at baseline. As a continuous smoking variable, we chose a cigarette-year that indicates the number of cigarettes per day multiplied by the number of years smoked. Moreover, we classified the participants as never-smokers, former smokers, and current smokers. Former smokers informed that they have not smoked within a month.

The KIHD continuous alcohol consumption variable indicates the amount of alcohol as grams per week. For this study, we categorized the participants into those with no health risk due to the alcohol consumption, one portion (12 grams of pure alcohol according to Finnish standards) per week at most, those with a moderate health risk, three portions per day at most, and those with a high health risk. This categorization is mainly data-specific, although it sparsely follows Finnish current care guidelines published only in Finnish. Broadly, alcohol increases mortality and morbidity and, in men, more than three to four portions, 40 grams of pure alcohol, per day increase them significantly [22].

To determine study participants’ physical activity we, first, calculated the basal energy expenditure (BEE) based a body weight, body height, and age applying the Mifflin-St Jeor Equation [23]. Second, we subtracted BEE from the total energy expenditure (TEE) and used this TEE − BEE variable in the analyses as a continuous variable. To create activity ranks, we computed the physical activity level (PAL) by dividing TEE by BEE and classified the participants as follows: moderately active, PAL < 2.00, vigorously active, PAL 2.00 − 2.40, and extremely active, PAL > 2.40 [24]. In the KIHD cohort, practically, all participants were at least moderately active at baseline. Eight participants of this study had not reported their physical activity.

Body weights and heights were not self-reported but measured by a research nurse during the baseline examination. Based on these measures we calculated the Body Mass Index (BMI) by dividing the weight in kilograms by the square of height in metres. In the analyses, we obeyed the standard guidelines for BMI: <25.0 kg/m^2^ refers to normal weight, 25.0−29.9 kg/m^2^ to overweight, and ≥30.0 kg/m^2^ to obesity [25] and classified the participants according to them.

On the first baseline examination day, one research nurse measured the participant’s blood pressure six times with a random-zero mercury sphygmomanometer. After a supine rest of five minutes, the nurse took three measurements in supine, two in sitting, and one in a standing position with 5-min intervals. In the present analyses, we used the mean of six systolic blood pressures (SBP) values as a continuous variable. To distribute study participants into groups according to SBP, we followed the thresholds suggested by Mayo Clinic: SBP < 120 mmHg is a desirable level and SBP > 139 mmHg indicates hypertension [26].

Study participants gave blood samples between 8 and 10 a.m. after abstaining from alcohol for three days and from smoking and eating for 12 h. After a supine rest of 30 min, a research nurse draw blood with Terumo Venoject VT-100PZ vacuum tubes (Terumo Corp., Tokyo, Japan) using no tourniquet. The laboratory of our institute used an enzymatic method to measure STC concentrations (CHOD-PAP, Boehringer Mannheim, Mannheim, West Germany) and a glucose dehydrogenase method (Merck, Darmstadt, West Germany) after protein precipitation with TCA using a clinical chemistry analyzer (Kone Specific, KONE Instruments Oy, Espoo, Finland) to measure FBG concentrations. Salonen *et al.* [27] describe the lipid analysis in detail. For the present analyses, we classified the participants according to the serum total cholesterol (SCT) as follows: <5.2 mmol/L is a desirable level and >6.2 mmol/L indicates hypercholesterolaemia [28]. Correspondingly, we distributed the participants into groups according to the fasting blood glucose (FBG) as follows: < 5.6 mmol/L is a desirable level and > 6.9 indicates diabetes [29].

### Statistical analyses

**Figure 1.**
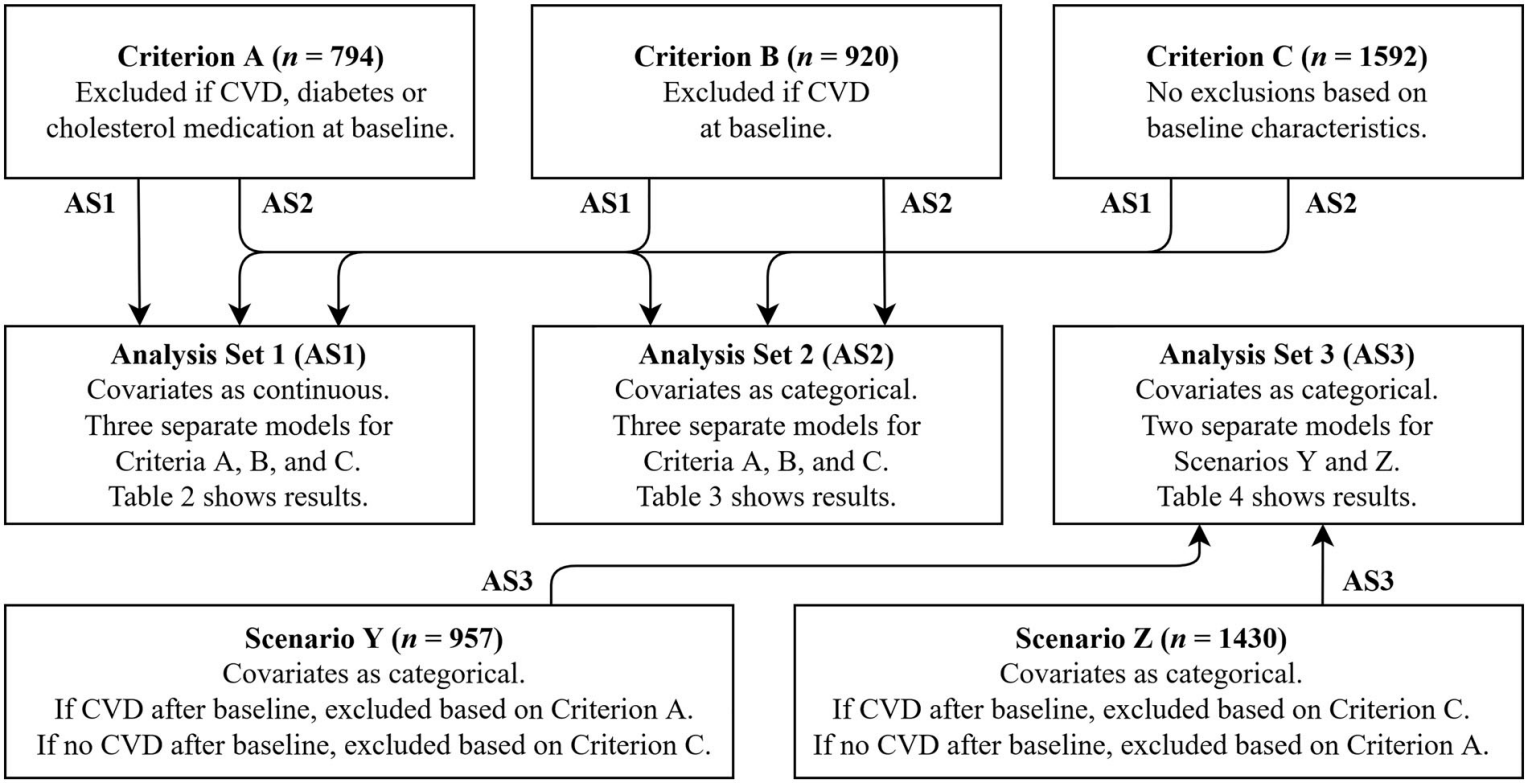
Procedure for statistical analyses. First, analysis sets (AS) 1 and 2 studied effects of participants exclusions on analysis results prospectively; the exclusions were based on baseline characteristics (Criteria A − C). Second, AS 1 and 2 studied effects of covariate manipulations (continuous vs. categorical) on analysis results. Third, AS 3 studied effects of participant exclusions on analysis results retrospectively; the exclusions were based on outcomes (Scenarios Y and Z). Fourth, all AS studied effects of outcome sensitivity on analysis results (see Tables 2–4).

The Cox proportional-hazards model [30] served as an analysis method and IBM® SPSS^®^ Statistics Version 25 served a statistical platform. In all analyses, we applied three different data exclusion criteria (Figure 1). The first criterion, termed as Criterion A later in the text, excluded study participants according to conditions. Precisely, we excluded participants, who reported that they have any CVD or diabetes at baseline or that they use hypercholesterolaemia medication. This exclusion criteria reduced the number of study participants from 1592 to 794. The second criterion, Criterion B, excluded study participants, who reported that they have a CVD, except for hypertension, at baseline. This criterion resulted in 920 participants. The third criterion, Criterion C, meant no exclusions. Correspondingly, in all analyses, we used CVD, CHD, AMI or UA, and a fatal AMI as dependent variables. These four “nested” outcomes demonstrate the outcome variable selection process with respect to outcome sensitivity. Moreover, to study the effect of covariate manipulations on the Cox model results, we executed Cox regressions adjusted for seven covariates, the six traditional risk factors and alcohol consumption that were either in their original continuous form or distributed in predetermined categories.

Altogether, we performed three analysis sets (Figure 1). The first set included covariates as continuous variables and tested their associations with CVD, CHD, AMI or UA, and a fatal AMI separately for each data exclusions criterion, A, B, and C. The second set included covariates as categorical variables. The reference categories were as follows: never-smoker, no health risk due to the alcohol consumption, normal weight, moderately physically active, desirable SBP, desirable FBG, and desirable STC. As the first set, the second set tested associations of covariates with CVD, CHD, AMI or UA, and a fatal AMI separately for each data exclusions criterion, A, B, and C. The third set, also, included covariates as categorical variables but used different data exclusion criteria for study participants, who received a CVD diagnosis during the follow-up, and for those, who did not.

The third analysis set constituted two analysis scenarios (Figure 1). In the first scenario, termed as Scenario Y later in the text, the exclusion of men with CVD during the follow-up was based on Criterion A and that of men with no CVD during the follow-up was based on Criterion C i.e. no exclusions. This resulted in 957 study participants eligible for the analysis. In the second scenario, Scenario Z, the exclusion of men with CVD during the follow-up was based on Criterion C and that of men with no CVD during the follow-up on Criterion A. Scenario Z resulted in 1430 study participants.

## Results

### Outcome sensitivity

CVD and a fatal AMI associated with covariates differently compared to each other as well as compared to CHD and MI or UA (Tables 2–4). CVD was the outcome that most evidently associated with SBP; a high SBP increased the risk of CVD. A fatal AMI in turn was the only outcome that showed only statistically non-significant associations with SBP and physical activity. CHD and MI or UA highlighted the same risk factors. Specifically, they associated with STC more strongly than CVD and a fatal AMI did.

**Table 2.** Hazard ratios and corresponding *p*-values of any cardiovascular disease (CVD), coronary heart disease (CHD), a myocardial infarction (MI) or unstable angina (UA), and a fatal acute myocardial infarction (AMI) with respect to one unit (1 U) or one standard deviation (1 D) increase in seven factors used as continues covariates in the Cox proportional-hazards model.

	CVD			CHD			MI or UA			AMI		
Covariate	1U	1D	*p*	1U	1D	*p*	1U	1D	*p*	1U	1D	*p*
A. Smoking^a^	**1.00**	**1.20**	**<.01**	**1.00**	**1.26**	**<.01**	**1.00**	**1.36**	**<.01**	1.00	1.27	.16
B. Smoking	**1.00**	**1.17**	**<.01**	**1.00**	**1.23**	**<.01**	**1.00**	**1.32**	**<.01**	1.00	1.18	.28
C. Smoking	**1.00**	**1.18**	**<.01**	**1.00**	**1.22**	**<.01**	**1.00**	**1.33**	**<.01**	**1.00**	**1.42**	**<.01**
A. AC^b^	1.00	1.05	.32	1.00	1.01	.89	1.00	1.01	.87	1.00	1.15	.39
B. AC	1.00	1.05	.30	1.00	1.00	.96	1.00	0.97	.68	1.00	1.11	.48
C. AC	1.00	1.00	.95	1.00	0.94	.24	1.00	0.95	.39	1.00	1.06	.58
A. BMI^c^	**1.03**	**1.12**	**.02**	1.04	1.13	.05	1.03	1.12	.14	1.08	1.29	.06
B. BMI	**1.04**	**1.14**	**<.01**	**1.05**	**1.19**	**<.01**	**1.05**	**1.19**	**.01**	**1.08**	**1.31**	**.04**
C. BMI	**1.05**	**1.20**	**<.01**	**1.05**	**1.20**	**<.01**	**1.05**	**1.21**	**<.01**	**1.08**	**1.32**	**<.01**
A. PAL^d^	1.00	0.99	.78	1.00	0.95	.43	1.00	0.96	.64	1.00	1.14	.42
B. PAL	1.00	0.98	.66	1.00	0.93	.25	1.00	0.94	.43	1.00	1.15	.36
C. PAL	**1.00**	**0.93**	**.04**	**1.00**	**0.90**	**.02**	**1.00**	**0.89**	**.02**	1.00	0.90	.28
A. SBP^e^	**1.01**	**1.18**	**<.01**	1.00	1.05	.44	1.00	1.03	.73	1.01	1.18	.32
B. SBP	**1.01**	**1.17**	**<.01**	1.01	1.09	.15	1.01	1.09	.21	1.01	1.24	.14
C. SBP	**1.01**	**1.18**	**<.01**	**1.01**	**1.10**	**.02**	**1.01**	**1.13**	**.01**	1.01	1.16	.09
A. FBG^f^	**1.19**	**1.13**	**.03**	**1.39**	**1.26**	**<.01**	**1.51**	**1.32**	**<.01**	**1.55**	**1.35**	**.01**
B. FBG	**1.18**	**1.19**	**<.01**	**1.14**	**1.15**	**<.01**	**1.19**	**1.20**	**<.01**	**1.25**	**1.27**	**.01**
C. FBG	**1.13**	**1.16**	**<.01**	**1.10**	**1.12**	**<.01**	**1.09**	**1.11**	**.01**	**1.12**	**1.15**	**.02**
A. STC^g^	1.04	1.04	.34	**1.17**	**1.18**	**.01**	**1.20**	**1.21**	**.01**	1.18	1.19	.30
B. STC	1.03	1.04	.42	**1.15**	**1.17**	**.01**	**1.22**	**1.24**	**<.01**	1.22	1.24	.11
C. STC	**1.08**	**1.09**	**.01**	**1.18**	**1.20**	**<.01**	**1.20**	**1.23**	**<.01**	**1.25**	**1.28**	**<.01**

*Note*. A refers to a dataset excluding CVD, diabetes, and high total cholesterol at baseline (*n* = 794). B refers to a dataset excluding CVD, except for hypertension, at baseline (*n* = 920). C refers to a dataset with no exclusions (*n* = 1592). Bold font indicates a statistically significant HR.

^a^ Cigarettes per day times years of smoking.

^b^ Alcohol consumption grams/week.

^c^ Body Mass Index, weight in kg divided by the square of height in m.

^d^ Physical activity level, total energy expenditure minus basal, in kcal per day.

^e^ Systolic blood pressure in mmHg.

^f^ Fasting blood glucose in mmol/L.

^g^ Serum total cholesterol in mmol/L.

### Participant exclusions

Cox regressions based on data with no exclusions most often discovered statistically significant associations of CHD with its risk factors, irrespective of covariate manipulations (Tables 2 and 3). In all these associations, the direction of the association was correct i.e. the risk factors related to hazard ratios (HR) larger than one and the protective factors related to HRs below one. Only regressions based on data with no exclusions identified, statistically significantly, the protective effect of physical activity; the highest category versus the lowest one. Appendix presents sample size calculations regarding the main outcome of this study, CHD, and Criterion A that excluded study participants according to conditions at baseline.

**Table 3.** Hazard ratios (HR), probabilities (P), and corresponding *p*-values of any cardiovascular disease (CVD), coronary heart disease (CHD), a myocardial infarction (MI) or unstable angina (UA), and a fatal acute myocardial infarction (AMI) with respect to seven factors used as categorical covariates in the Cox proportional-hazards model.

	CVD			CHD			MI or UA			AMI		
Covariate	HR	P	*p*	HR	P	*p*	HR	P	*p*	HR	P	*p*
A. Former smoker	1.07	0.52	.53	1.15	0.53	.35	**1.49**	**0.60**	**.03**	0.76	0.43	.51
B. Former smoker	1.11	0.53	.30	1.19	0.54	.20	**1.42**	**0.59**	**.04**	0.72	0.42	.38
C. Former smoker	**1.19**	**0.54**	**.02**	**1.24**	**0.55**	**.02**	**1.40**	**0.58**	**<.01**	1.09	0.52	.71
A. Current smoker	**1.50**	**0.60**	**<.01**	**1.78**	**0.64**	**<.01**	**2.31**	**0.70**	**<.01**	**2.36**	**0.70**	**.03**
B. Current smoker	**1.49**	**0.60**	**<.01**	**1.64**	**0.62**	**<.01**	**2.05**	**0.67**	**<.01**	**2.00**	**0.67**	**.04**
C. Current smoker	**1.50**	**0.60**	**<.01**	**1.69**	**0.63**	**<.01**	**1.94**	**0.66**	**<.01**	**2.74**	**0.73**	**<.01**
A. AC^a^ 13 − 252	1.05	0.51	.64	0.95	0.49	.68	0.98	0.49	.92	0.63	0.39	.18
B. AC 13 − 252	1.04	0.51	.68	0.90	0.47	.35	0.97	0.49	.82	0.84	0.46	.58
C. AC 13 − 252	1.03	0.51	.64	0.90	0.47	.17	0.96	0.49	.66	0.80	0.44	.22
A. AC >252	1.29	0.56	.30	1.13	0.53	.73	1.05	0.51	.92	1.48	0.60	.60
B. AC >252	1.20	0.55	.41	0.94	0.48	.85	0.86	0.46	.72	1.19	0.54	.82
C. AC >252	1.16	0.54	.31	0.91	0.48	.64	0.98	0.49	.93	1.23	0.55	.59
A. BMI^b^ 25.0 − 29.9	1.14	0.53	.20	1.19	0.54	.21	1.33	0.57	.09	0.90	0.47	.78
B. BMI 25.0 − 29.9	1.18	0.54	.07	1.27	0.56	.06	**1.41**	**0.59**	**.03**	0.93	0.48	.84
C. BMI 25.0 − 29.9	**1.26**	**0.56**	**<.01**	**1.42**	**0.59**	**<.01**	**1.49**	**0.60**	**<.01**	1.04	0.51	.86
A. BMI ≥30.0	**1.41**	**0.59**	**.02**	**1.51**	**0.60**	**.03**	1.47	0.60	.12	**2.85**	**0.74**	**.02**
B. BMI ≥30.0	**1.54**	**0.61**	**<.01**	**1.64**	**0.62**	**.01**	**1.70**	**0.63**	**.02**	**2.51**	**0.72**	**.02**
C. BMI ≥30.0	**1.75**	**0.64**	**<.01**	**1.79**	**0.64**	**<.01**	**1.90**	**0.66**	**<.01**	**2.37**	**0.70**	**<.01**
A. PAL^c^ Vigorous	1.05	0.51	.73	0.94	0.48	.73	0.93	0.48	.74	0.79	0.44	.63
B. PAL Vigorous	1.07	0.52	.59	0.98	0.49	.89	0.99	0.50	.96	1.14	0.53	.78
C. PAL Vigorous	0.91	0.48	.24	0.86	0.46	.17	0.84	0.46	.19	0.72	0.42	.18
A. PAL Extreme	1.02	0.50	.85	0.89	0.47	.46	0.89	0.47	.56	1.06	0.51	.90
B. PAL Extreme	1.03	0.51	.79	0.87	0.47	.34	0.89	0.47	.52	1.31	0.57	.54
C. PAL Extreme	**0.84**	**0.46**	**.03**	**0.78**	**0.44**	**.01**	**0.74**	**0.43**	**.02**	0.72	0.42	.16
A. SBP^d^ 120 − 139	1.11	0.53	.38	0.99	0.50	.96	0.89	0.47	.57	1.98	0.66	.22
B. SBP 120 − 139	1.16	0.54	.20	1.02	0.50	.90	0.87	0.47	.45	2.26	0.69	.13
C. SBP 120 − 139	1.12	0.53	.20	0.96	0.49	.68	0.84	0.46	.18	1.24	0.55	.44
A. SBP ≥140	**1.50**	**0.60**	**<.01**	1.17	0.54	.39	1.02	0.50	.95	2.15	0.68	.19
B. SBP ≥140	**1.50**	**0.60**	**<.01**	1.20	0.55	.27	1.06	0.51	.79	2.71	0.73	.07
C. SBP ≥140	**1.42**	**0.59**	**<.01**	1.13	0.53	.28	1.09	0.52	.52	1.48	0.60	.18
A. FBG^e^ 5.6 − 6.9	1.51	0.60	.07	1.67	0.63	.08	1.91	0.66	.05	0.78	0.44	.81
B. FBG 5.6 − 6.9	1.40	0.58	.08	**1.68**	**0.63**	**.03**	**2.11**	**0.68**	**.01**	1.12	0.53	.88
C. FBG 5.6 − 6.9	**1.30**	**0.57**	**.04**	**1.73**	**0.63**	**<.01**	**1.78**	**0.64**	**<.01**	**2.05**	**0.67**	**.02**
A. FBG >6.9	**3.07**	**0.75**	**<.01**	**7.21**	**0.88**	**<.01**	**5.29**	**0.84**	**<.01**	2.95	0.75	.16
B. FBG >6.9	**3.03**	**0.75**	**<.01**	**3.64**	**0.78**	**<.01**	**3.90**	**0.80**	**<.01**	2.73	0.73	.07
C. FBG >6.9	**2.53**	**0.72**	**<.01**	**2.61**	**0.72**	**<.01**	**2.28**	**0.70**	**<.01**	**2.95**	**0.75**	**<.01**
A. STC^f^ 5.2 − 6.2	1.16	0.54	.20	**1.42**	**0.59**	**.04**	1.32	0.57	.19	1.41	0.59	.47
B. STC 5.2 − 6.2	1.07	0.52	.52	1.23	0.55	.16	1.17	0.54	.38	0.91	0.48	.82
C. STC 5.2 − 6.2	1.09	0.52	.29	**1.24**	**0.55**	**.03**	**1.35**	**0.57**	**.02**	1.21	0.55	.45
A. STC >6.2	1.19	0.54	.14	**1.47**	**0.60**	**.02**	**1.57**	**0.61**	**.03**	1.91	0.66	.15
B. STC >6.2	1.07	0.52	.52	1.27	0.56	.10	**1.41**	**0.59**	**.04**	1.48	0.60	.28
C. STC >6.2	**1.20**	**0.55**	**.02**	**1.39**	**0.58**	**<.01**	**1.56**	**0.61**	**<.01**	**1.62**	**0.62**	**.04**

*Note*. A refers to a dataset excluding CVD, diabetes, and high total cholesterol at baseline (*n* = 794). B refers to a dataset excluding CVD, except for hypertension, at baseline (*n* = 920). C refers to a dataset with no exclusions (*n* = 1592). Bold font indicates a statistically significant HR.

^a^ Alcohol consumption in g/week.

^b^ Body Mass Index, kg/m^2^.

^c^ Physical activity level, the total energy expenditure divided by the basal energy expenditure, moderate <2.00, extreme >2.40.

^d^ Systolic blood pressure in mmHg.

^e^ Fasting blood glucose in mmol/L.

^f^ Serum total cholesterol in mmol/L.

The comparison between Scenarios Y and Z showed that strict data exclusions regarding men with no CVD during the follow-up combined with no exclusions regarding men with CVD during the follow-up yielded more often statistically significant and plausible results than no data exclusions concerning men with no CVD and strict exclusions regarding men with CVD (Table 4).

**Table 4.** Hazard ratios (HR), probabilities (P), and corresponding *p*-values of any cardiovascular disease (CVD), coronary heart disease (CHD), a myocardial infarction (MI) or unstable angina (UA), and a fatal acute myocardial infarction (AMI) with respect to seven factors used as categorical covariates in the Cox proportional-hazards model.

	CVD			CHD			MI or UA			AMI		
Covariate	HR	P	*p*	HR	P	*p*	HR	P	*p*	HR	P	*p*
Y. Former smoker	1.06	0.51	.56	1.01	0.50	.52	1.37	0.58	.09	0.75	0.43	.48
Z. Former smoker	**1.18**	**0.54**	**.02**	**1.25**	**0.56**	**.02**	**1.45**	**0.59**	**<.01**	1.17	0.54	.52
Y. Current smoker	**1.36**	**0.58**	**.01**	**1.56**	**0.61**	**<.01**	**1.96**	**0.66**	**<.01**	1.96	0.66	.07
Z. Current smoker	**1.59**	**0.61**	**<.01**	**1.79**	**0.64**	**<.01**	**2.14**	**0.68**	**<.01**	**3.16**	**0.76**	**<.01**
Y. AC^a^ 13 − 252	1.08	0.52	.42	0.96	0.49	.75	1.03	0.51	.88	0.69	0.41	.26
Z. AC 13 − 252	1.00	0.50	.98	0.89	0.47	.14	0.90	0.47	.31	0.73	0.42	.10
Y. AC > 252	1.22	0.55	.42	0.86	0.46	.67	0.87	0.47	.74	1.25	0.56	.77
Z. AC > 252	1.19	0.54	.24	1.13	0.53	.52	1.16	0.54	.53	1.42	0.59	.37
Y. BMI^b^ 25.0 − 29.9	1.12	0.53	.23	1.13	0.53	.36	1.21	0.55	.26	0.83	0.45	.62
Z. BMI 25.0 − 29.9	**1.25**	**0.56**	**<.01**	**1.42**	**0.59**	**<.01**	**1.55**	**0.61**	**<.01**	1.13	0.53	.59
Y. BMI ≥ 30.0	**1.46**	**0.59**	**.01**	1.23	0.55	.29	1.17	0.54	.52	1.97	0.66	.13
Z. BMI ≥ 30.0	**1.66**	**0.62**	**<.01**	**1.94**	**0.66**	**<.01**	**2.16**	**0.68**	**<.01**	**3.08**	**0.75**	**<.01**
Y. PAL^c^ Vigorous	1.01	0.50	.95	0.94	0.48	.70	0.92	0.48	.68	0.82	0.45	.68
Z. PAL Vigorous	0.92	0.48	.32	0.87	0.47	.20	0.85	0.46	.20	0.71	0.42	.16
Y. PAL Extreme	0.97	0.49	.83	0.87	047	.41	0.86	0.46	.44	1.09	0.52	.85
Z. PAL Extreme	0.86	0.46	.07	**0.79**	**0.44**	**.02**	**0.76**	**0.43**	**.02**	0.70	0.41	.12
Y. SBP^d^ 120 − 139	1.16	0.54	.21	0.97	0.49	.84	0.86	0.46	.46	1.97	0.66	.22
Z. SBP 120 − 139	1.08	0.52	.36	0.98	0.49	.86	0.88	0.47	.33	1.30	0.57	.35
Y. SBP ≥ 140	**1.57**	**0.61**	**<.01**	1.19	0.54	.33	1.05	0.51	.82	2.02	0.67	.22
Z. SBP ≥ 140	**1.38**	**0.58**	**<.01**	1.14	0.53	.26	1.09	0.52	.54	1.54	0.61	.14
Y. FBG^e^ 5.6 − 6.9	1.12	0.53	.61	1.34	0.57	.31	1.46	0.59	.25	0.57	0.36	.59
Z. FBG 5.6 − 6.9	**1.52**	**0.60**	**<.01**	**1.93**	**0.66**	**<.01**	**2.15**	**0.68**	**<.01**	**2.55**	**0.72**	**<.01**
Y. FBG > 6.9	1.86	0.65	.07	**2.86**	**0.74**	**<.01**	**2.21**	**0.69**	**.04**	1.89	0.65	.39
Z. FBG > 6.9	**2.96**	**0.75**	**<.01**	**3.27**	**0.77**	**<.01**	**2.99**	**0.75**	**<.01**	**4.36**	**0.81**	**<.01**
Y. STC^f^ 5.2 − 6.2	1.19	0.54	.14	**1.49**	**0.60**	**.02**	1.48	0.60	.06	1.50	0.60	.38
Z. STC 5.2 − 6.2	1.07	0.52	.36	**1.26**	**0.56**	**.02**	1.25	0.56	.08	1.11	0.53	.68
Y. STC > 6.2	1.20	0.55	.12	**1.48**	**0.60**	**.02**	**1.66**	**0.62**	**.01**	1.93	0.66	.14
Z. STC > 6.2	**1.20**	**0.55**	**.02**	**1.43**	**0.59**	**<.01**	**1.50**	**0.60**	**<.01**	1.54	0.61	.07

*Note*. Y refers to a dataset with no exclusions for study participants with no CVD during the follow-up (*n* = 411) and excluding CVD, diabetes, and high total cholesterol at baseline for study participants with CVD during the follow-up (*n* = 546). Z refers to a dataset excluding CVD, diabetes, and high total cholesterol at baseline for study participants with no CVD during the follow-up (*n* = 248) and no exclusions for study participants with CVD during the follow-up (*n* = 1182). Bold font indicates a statistically significant HR.

^a^ Alcohol consumption (g/week).

^b^ Body Mass Index (kg/m^2^).

^c^ Physical activity level, the total energy expenditure divided by the basal energy expenditure, moderate <2.00, extreme >2.40.

^d^ Systolic blood pressure (mmHg).

^e^ Fasting blood glucose (mmol/L).

^f^ Serum total cholesterol (mmol/L).

### Covariate manipulations

There were only minor differences in Cox model results between analyses including covariates as continuous variables and those including covariates as categorical variables (Tables 2–4). Continuous and categorical covariates led to the same conclusions regarding the association of CHD with its risk factors. Being a former or current smoker, being overweight or obese, and having borderline high or high FBG or STC levels significantly increased the risk of CHD. The effect of high SBP levels on the risk of CHD was uncertain as well as the protective effect of physical activity. Our analyses found no statistically significant association between CHD and alcohol consumption.

## Discussion

Traditionally, epidemiological studies use in their analyses only study participants who are free of the disease of interest at baseline. Our study suggests that excluding study participants who have the disease already at baseline is probably unnecessary. Specifically, our analyses led to the best results when we included all study participants who received the diagnosis during the follow-up irrespective of their self-reported baseline statuses but excluded all study participants who did not receive the diagnosis during the follow-up but had self-reported the disease at baseline. Moreover, our study does not, unconditionally, support participant exclusions with respect to covariates either. Excluding participants who are at risk already at baseline may enable discovering the strongest associations, such as the relationship between diabetes and CHD, but, simultaneously, it may fade out weaker, although relevant, associations, such as the relationship between physical activity and CHD. In other words, a combination of “loose cases” and “strict controls” may yield the best results. In the next paragraphs, we evaluate our results from the viewpoint of CHD risk factors.

In our study, smoking, overweight, and high blood glucose levels, evidently, associated with CHD. Outcome variable selection, participant exclusion, and covariate manipulation procedures had no effects on conclusions drawn from results related to these three cornerstone risk factors. Being a current smoker or being obese (BMI ≥ 30.0) resulted in 1.5 times higher hazard compared to never smokers and normal-weight study participants, whereas diabetes (FBG > 6.9 mmol/L) approximately tripled the hazard of CHD. Large prospective cohort studies have reported even stronger effects of smoking and obesity on CHD already in 1960s [31]. The three times higher hazard of CHD among diabetic men seems to be a rule of thumb [9].

Total cholesterol and blood pressure were the covariates that most evidently revealed differences related to outcome sensitivity. Total cholesterol is only one of many measures of the lipid status of which all show somewhat unique associations with CHD and other CVDs [10,13]. Total cholesterol, for example, does not associate as strongly with the risk of stroke [32] as it associates with the risk of CHD [10]. Conversely, high blood pressure increases, specifically, the risk of stroke [33], which may for its part explain, together with reasons related to the sample size, why SBP associated statistically significantly with CVD but not with CHD and MIs in our study.

Irrespective of outcome variable selection, participant exclusions, and covariate manipulations, our study found no statistically significant effects of alcohol consumption on the hazard of CHD. Although alcohol, in general, increases mortality and morbidity [22], light-to-moderate drinking may protect against CHD [11], which for its part may complicate the statistical detection of the association between alcohol consumption and CHD. Moreover, the association relates to the pattern of consumption i.e. binge drinking *via* the progression of atherosclerosis [34], which we did not considered in this study.

## Limitations

Our results are based on one dataset and, therefore, them are not straightforwardly generalizable. Moreover, our study does not consider severity of diseases per se or diagnoses other than CVD, CHD, MI or UA, and AMI.

All KIHD study participants, practically, were at least moderately active at baseline and nearly half of them were extremely active based on PAL values. This indicates the active lifestyles of the KIHD study participants; many of them were farmers or lumberjacks and highly interested in cross-country skiing, which to some extent distinguishes the KIHD cohort from otherwise similar cohorts. On the other hand, extreme physical activity levels, PAL > 2.4, are unrealistic in the long run because they lead to a negative energy balance i.e. weight loss [35]. This contradiction, most probably, is due to the KIHD assessment method of physical activity. In general, self-assessment physical activity questionnaires show low validity and reliability [36]. Consequently, the present TEE and PAL values are adequate for creating data-specific activity ranks [37] but not for comparing the KIHD cohort to other cohorts as such.

## Conclusions

Our Cox model example of the epidemiological relationship between CHD and its common risk factors evidently demonstrated that outcome variable selection and participant exclusions must be considered when interpreting results of epidemiological analyses. Preprocessing procedures that were loose regarding study participants with any CVD during the follow-up and strict concerning study participants with no CVD during the follow-up were best in discovering the association between risk factors and CHD. Outcome sensitivity affected associations across covariates and outcomes. For example, total cholesterol associated, specifically, with CHD and MI or UA but weakly with CVD or AMI. The covariate type, continuous or categorial, had only minor effects on Cox model results. We strongly suggest that research reports present results based on no data exclusions together with results based on reasoned exclusions.

## Data Availability

On request, the University of Eastern Finland’s Institute of Public Health and Clinical Nutrition can admit an access to the KIHD database.

## Appendix

**Table A1.** Results of sample size calculations regarding the main outcome of this study, coronary heart disease (CHD), with respect to Criterion A that excluded study participants according to conditions at baseline.

	Observed			Required *n*	
	*n*	CHD *n* (%)	No CHD *n* (%)		
Smoking: Never-smoker (Reference)	296	100 (34)	196 (66)	4213	1140
Smoking: Former smoker	270	101 (37)	169 (63)	3792	
Smoking: Current smoker	228	92 (40)	136 (60)		912
Alcohol consumption: Light	308	112 (36)	196 (64)		2854
Alcohol consumption: Moderate	454	172 (38)	282 (62)	1843	
Alcohol consumption: Heavy (Reference)	32	9 (28)	23 (72)	184	285
Normal weight (Reference)	278	94 (34)	184 (66)	1886	794
Overweight	415	156 (38)	259 (62)	2829	
Obese	101	43 (43)	58 (57)		318
Physical activity: Moderate	144	55 (38)	89 (62)		6390
Physical activity: Vigorous	251	93 (37)	158 (63)	29089	
Physical activity: Extreme (Reference)	391	141 (36)	250 (64)	48481	15975
Systolic blood pressure: Desirable	141	52 (37)	89 (63)	5860	
Systolic blood pressure: Prehypertension (Reference)	404	140 (35)	264 (65)	19535	1364
Systolic blood pressure: Hypertension	249	101 (41)	148 (59)		818
Fasting blood glucose: Desirable (Reference)	753	269 (36)	484 (64)	5945	239
Fasting blood glucose: Prediabetes	31	14 (45)	17 (55)	238	
Fasting blood glucose: Diabetes	10	10 (100)	0 (0)		2
Serum total cholesterol: Desirable (Reference)	190	55 (29)	135 (71)	432	177
Serum total cholesterol: Pre-hypercholesterolaemia	315	118 (37)	197 (63)	734	
Serum total cholesterol: Hypercholesterolaemia	289	120 (42)	169 (58)		266

*Note*. Required *n* refers to comparisons across the category in which the proportion of CHD diagnoses during the follow-up was lowest (reference) and other categories applying a predetermined *p*-value of .05 (α) and a predetermined power of 0.80 (1 − β).

***An example of sample size calculations: serum total cholesterol***

$$n_{1}=\frac{{\left[z_{1-\frac{\alpha }{2}}\times \sqrt{\bar{p}\times \bar{q}\times (1+\frac{1}{k})}+\;z_{1-\beta }\;\times \sqrt{p_{1}\times q_{1}+(\frac{p_{2}\times q_{2}}{k})}\right]}^{2}}{\Delta^{2}}$$

$$q_{1}=1-p_{1}$$

$$q_{2}=1-p_{2},$$

$$\bar{p}=\frac{p_{1}+{kp}_{2}}{1+k},$$

$$\bar{q}=1-\bar{p}.$$

$n_{1}$ = The number of participants in Group 1
$n_{2}$ = The number of participants in Group 2.
α = The probability of Type I error
β = The probability of Type II error.
z = The standardized value re the predetermined α and β.
k = The ratio of $n_{2}$ to $n_{1}.$
$p_{1}$ = The proportion of participants with a follow-up CHD diagnose in Group 1.
$p_{2}$ = The proportion of participants with a follow-up CHD diagnose in Group 2.

$$n_{1}=\frac{{\left[1.96\;\times \sqrt{0.368\times 0.632\;\times \;(1+\frac{1}{1.5})}+0.84\;\times \sqrt{0.29\times 0.71\;+\;(\frac{0.42\times 0.58}{1.5})}\right]}^{2}}{0.13^{2}}=177$$

$n_{2}=k\times n_{1}=266.$

**References**

Rosner B. *Fundamentals of Biostatistics*. 8th ed. Boston (MA): CENGAGE Learning; 2016.
